# Streamlining recombination-mediated genetic engineering by validating three neutral integration sites in *Synechococcus* sp. PCC 7002

**DOI:** 10.1186/s13036-017-0061-8

**Published:** 2017-06-05

**Authors:** Anne Ilse Maria Vogel, Rahmi Lale, Martin Frank Hohmann-Marriott

**Affiliations:** Department of Biotechnology, PhotoSynLab, NTNU, Norwegian University of Science and Technology, Trondheim, Norway

**Keywords:** *Synechococcus* sp. PCC7002, Neutral integration sites, Genetic engineering, Transformation, BioBrick, Cyanobacteria, Synthetic biology

## Abstract

**Background:**

*Synechococcus* sp. PCC 7002 (henceforth *Synechococcus*) is developing into a powerful synthetic biology chassis. In order to streamline the integration of genes into the *Synechococcus* chromosome, validation of neutral integration sites with optimization of the DNA transformation protocol parameters is necessary. Availability of BioBrick-compatible integration modules is desirable to further simplifying chromosomal integrations.

**Results:**

We designed three BioBrick-compatible genetic modules, each targeting a separate neutral integration site, A2842, A0935, and A0159, with varying length of homologous region, spanning from 100 to 800 nt. The performance of the different modules for achieving DNA integration were tested. Our results demonstrate that 100 nt homologous regions are sufficient for inserting a 1 kb DNA fragment into the *Synechococcus* chromosome. By adapting a transformation protocol from a related cyanobacterium, we shortened the transformation procedure for *Synechococcus* significantly.

**Conclusions:**

The optimized transformation protocol reported in this study provides an efficient way to perform genetic engineering in *Synechococcus*. We demonstrated that homologous regions of 100 nt are sufficient for inserting a 1 kb DNA fragment into the three tested neutral integration sites. Integration at A2842, A0935 and A0159 results in only a minimal fitness cost for the chassis. This study contributes to developing *Synechococcus* as the prominent chassis for future synthetic biology applications.

**Electronic supplementary material:**

The online version of this article (doi:10.1186/s13036-017-0061-8) contains supplementary material, which is available to authorized users.

## Background

The ability of cyanobacteria to utilize sunlight for capturing CO_2_ makes them powerful cell factories [1]. Cyanobacteria can be genetically engineered to produce industrially relevant chemicals such as isobutanol [2], sucrose [3], hydrogen [4] and ethylene [5]. When choosing a cyanobacterium as a chassis, genetic amenability and growth characteristics are important criteria. Several cyanobacterial strains are already available which possess promising traits for biotechnology.

*Synechocystis* sp. PCC 6803 (henceforth *Synechocystis*) is the most frequently investigated cyanobacterium and several genetic tools have been designed for effective metabolic engineering [6]. However, *Synechocystis* has a relatively slow doubling time, minimally 8 h [7], and prefers freshwater medium for growth, limiting its’ potential as a biotechnological platform.

The halotolerant cyanobacterium *Synechococcus* sp. PCC 7002 (formerly known as *Agmenellum quadruplicatum* strain PR-6, henceforth *Synechococcus*) is an excellent chassis for biotechnological applications. *Synechococcus* can utilize high-light irradiation, hence enabling *Synechococcus* to grow with a short doubling time of under 3 h [8–10]. Furthermore, *Synechococcus* can grow photoautotrophically, mixotrophically or heterotrophically and tolerates a wide range of temperatures and salt concentrations [8, 9, 11–13].

The interest in developing *Synechococcus* as a chassis in biotechnological applications is reflected by recent efforts to develop advanced genetic tools for this organism. A promoter library with IPTG or anhydrotetracycline-inducible gene cassettes is available, as well as an endogenous plasmid-based system for gene overexpression [14–16]. Additionally, a fluorescent protein reporter system has been developed for *Synechococcus* [17]. With the advancements of synthetic biology, the BioBrick system [18] could also be utilized in *Synechococcus*.

A key advantage of *Synechococcus* is the ease of genetic engineering since *Synechococcus* can naturally take up linear double-stranded DNA (dsDNA) and incorporate dsDNA in its genome via homologous recombination [19]. Several transformation protocols for *Synechococcus* have become available with varying transformation efficiency [19–22]. However, the lengths of homologous regions required for efficient chromosomal integration has only been recently investigated for one chromosomal site [17]. The optimization of the length of homologous regions is a critical parameter for two reasons; (i) it directly influences the success rate of chromosomal integration and (ii) the cost of synthetic DNA increases exponentially with the length of synthesized DNA.

Suitable neutral integration sites (NISs) for standardized integration of non-native genes are an important tool for efficient genomic engineering. Several separate studies have focused on annotating possible NISs [23–25] in *Synechococcus* and the need for validation of NISs that are used frequently in *Synechococcus* has been made clear [17]. Although Ruffing et al. recently investigated the influence of the length of homologous regions on transformation efficiency at one NIS; no systematic studies are available on optimizing transformations targeting other often-used NISs in *Synechococcus* [17].

In this study, we study systematically how the length of homologous DNA regions affects the efficiency of chromosomal integrations in *Synechococcus* sp. PCC 7002 by targeting three previously annotated NISs. We generated three chromosomal integration modules carrying BioBrick Prefix and Suffix suitable for BioBrick-based cloning, forming the basis for successful synthetic biology approaches in *Synechococcus*.

Our results form the basis of a low-cost, high-performance transformation procedure in *Synechococcus*.

## Methods

### Organisms and maintenance culture conditions

The glycerol-utilizing strain *Synechococcus* sp. PCC 7002 was provided by Niels-Ulrik Frigaard (University of Copenhagen). *Synechococcus* wildtype and constructed mutants were maintained in a modified, Tris-buffered (pH 8.2) A+ medium derivative [19, 26], designated AA+. Instead of a P1 trace metal solution, an almost identical 1000× BG-11 trace mineral solution [20] was used. The 1000× BG-11 trace mineral solution was composed of 2.860 g/L H_3_BO_3_, 1.810 g/L MnCl_2_ · 4H_2_O, 0.222 g/L ZnSO_4_ · 7H_2_O, 0.390 g/L Na_2_MoO_4_ · 2H_2_O, 0.079 g/L CuSO_4_ · 5H_2_O and 0.0494 g/L Co (NO_3_) _2_ · 6H_2_O. The AA+ medium protocol including the preparation of stock solutions is available at the Benchling Protocol and Data Repository [27].

*Synechococcus* was grown in the presence of 15 μM glycerol. Liquid cultures of *Synechococcus* were grown in modified 500 mL Erlenmeyer flasks with light and temperature regimes as indicated. Aeration was provided by bubbling filtered air through ddH_2_O into liquid cultures with air pumps. Growth was monitored by measuring the optical density at 730 nm (OD_730_) with a SPECTRONIC 200 E spectrophotometer (Thermo Scientific). For solid medium, AA+ medium was supplemented with 1.5% agar-agar (Merck Millipore). Cells were grown on plates with AA+ medium under constant illumination and temperature as indicated. Kanamycin was used for the selection of kanamycin resistant mutants (50 μg/mL).

*Escherichia coli* strain DH5α was used for cloning and plasmid amplification. *E. coli* was grown at 37 °C in LB medium (10 g/L tryptone, 5 g/L yeast extract, 5 g/L NaCl) in culture tubes or on agar plates. Kanamycin (50 μg/mL) or ampicillin (100 μg/mL) was added when applicable.

### Plasmid construction

**Fig. 1:**
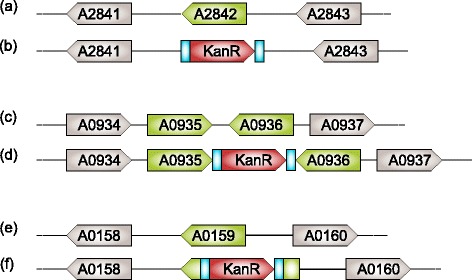
Schematic representation of the construction of neutral integration site strains. The genes in *green arrows* represent the genetic sites of interest. The BioBrick Prefix and Suffix are indicated in *blue* and the kanamycin resistance cassette in *red*. **a**, **c**, **e** Schematic representation of the genetic organization of wildtype chromosomal DNA *Synechococcus* sp. PCC 7002 at three NISs (not to scale). **b** Chromosomal DNA after homologous recombination replacing *SYNPCC7002_A2842* with a kanamycin resistance cassette flanked by the BioBrick Prefix and Suffix. **d** Chromosomal DNA after homologous recombination inserting a kanamycin resistance cassette flanked by the BioBrick Prefix and Suffix between *SYNPCC7002_A0935* and *SYNPCC7002_A0936*. **f** Chromosomal DNA after homologous recombination resulting in a disruption of *SYNPCC7002_A0159* by inserting a kanamycin resistance cassette flanked by the BioBrick Prefix and Suffix

Three potential NISs were selected based on previous studies [23–25]; henceforth called A2842, A0935 and A0159 (see Fig. 1). Plasmids were designed with Clone Manager Professional v9 (Sci-Ed Software) and with the online platform Benchling. The plasmids used as template for production of linear dsDNA fragments consisted of a pUC57-Simple backbone, the homologous regions 800 nt upstream and downstream of the NIS and an insertion of the BioBrick Prefix and BioBrick Suffix [18] between the upstream and downstream homologous region. Plasmids were ordered from Genscript and named pUC57Simple_A2842, pUC57Simple_A0935 and pUC57Simple_A0159. A BioBrick compatible kanamycin resistance cassette was inserted as described in the BioBrick Standard Assembly protocols [28]. The constructed plasmids were referred to as pUC57Simple_A2842Kan, pUC57Simple_A0935Kan and pUC57Simple_A0159Kan. Plasmid maps are available at the Benchling Plasmid Repository [29] and are displayed in the Additional file 1: Figure S1, Additional file 2: Figure S2 and Additional file 3: Figure S3.

### Construction of linear dsDNA fragments

Linear dsDNA fragments with either 800, 400, 200 or 100 nt homologous regions upstream and downstream of the targeted NISs were created by standard Q5High-Fidelity DNA Polymerase PCR (NEB). Either pUC57Simple_A2842Kan, pUC57Simple_A0935Kan or pUC57Simple_A0159Kan was used as template with designated primers (Additional file 4: Table S1). The correct length of PCR products was confirmed on 0.8% agarose gel and linear dsDNA fragments were purified with QIAquick PCR Purification Kit (Qiagen). Linear dsDNA fragments were diluted to a final concentration of 75 ng/μL.

### Transformation of *Synechococcus* sp. PCC 7002

Liquid cultures of *Synechococcus* were grown to an OD_730_ of 0.4. Cells were pelleted by centrifugation at 2500 x g for 8 min at 25 °C followed by suspending in AA+ medium to an OD_730_ of 8. Cells were aliquoted and 1 μg linear dsDNA was added to reach a total volume of 100 μL. Cultures were maintained for 6 h under constant temperature (30 °C) and low illumination (8 μE m^−2^ s^−1^). A full protocol including preparation of chemicals and calculations is available at the Benchling Protocol and Data Repository [27].

An aliquot of the transformed culture (100 μL for OD_730_-based analysis, 5 μL for camera-based analysis) was plated onto sterile membranes (Nuclepore Track-Etch membrane 0.4 μm, Whatman) positioned on non-selective AA+ plates. Plates were incubated for 16 h at 30 °C and under constant low illumination (8 μE m^−2^ s^−1^). The membranes were transferred to selective AA+ plates and incubated at 30 °C under constant low light (8 μE m^−2^ s^−1^) for 24 h. Plates were then transferred to 33 °C under constant high illumination (75 μE m^−2^ s^−1^) and transformation efficiency was analyzed by two approaches.

### Spectrophotometry-based transformation analysis

After 7 days under high illumination on selective plates, all colonies were scraped off from the membranes, suspended in 1 mL selective AA+ medium (50 μg/mL kanamycin) and quantified by OD_730_ measurements. For each strain, 2 μL were used for subsequent strain generation. Experiments were performed in triplicate.

### Camera-based transformation analysis

Quantitative images of each selective AA+ plate (50 μg/mL kanamycin) under high illumination were taken during 12 days using a custom-built plate imaging system. Experiments were performed in triplicate. The intensity of the spots was calculated and data were analyzed with the software Prism version 6 (GraphPad).

### Segregation of transformant *Synechococcus* strains

Prospective transformants were restreaked on selective AA+ plates (50 μg/mL kanamycin) and incubated at 33 °C under constant illumination (75 μE m^−2^ s^−1^). Full segregation was reached after restreaking twice within an 8-day incubation period. Full segregation was confirmed by colony PCR (Q5High-Fidelity DNA Polymerase) with primers aligning in the 800 nt homologous regions for A2842, A0935 and A0159. PCR products were visualized on 0.8% agarose gel. A wildtype strain of *Synechococcus* was used as a control for the PCR.

### Comparative growth experiments

Fully segregated *Synechococcus* strains A2842, A0935 and A0159 were grown in Erlenmyer flasks, by bubbling filtered air through ddH2O, with liquid AA+ medium supplemented with kanamycin until an OD_730_ of 0.8. Growth of the different strains was studied in liquid and on solid AA+ medium.

For studying growth in liquid AA+ medium, an aliquot of 1 mL of the cultures was washed three times with 1 mL AA+ medium and 40 μL of the cell suspension were transferred to culture flasks containing 40 mL non-selective AA+ medium. Cells were incubated in flasks at 33 °C, under constant agitation (150 rpm) and illumination (75 μE m^−2^ s^−1^). Growth was monitored by spectrophotometric measurements at OD_730_ every 24 h for 12 days.

For studying growth on solid AA+ medium, cultures were diluted 100 times and 2 μL of each culture was spotted on non-selective plates in triplicate. Plates were incubated at 33 °C with constant illumination (75 μE m^−2^ s^−1^). Quantitative images of plates were taken every 12 h for 9 days by using a custom-built plate imaging system and the change in intensity of the spots was calculated. Both experiments were performed in triplicate and a wildtype strain of *Synechococcus* was used as control. Raw data was used to calculate growth rates with the grofit package (logistic growth) in R as described before [30, 31]. Growth rates were normalized as percentage growth density of the wildtype and visualized with the software Prism version 6 (GraphPad).

## Results and discussion

### Neutral integration sites in *Synechococcus* sp. PCC 7002

In order to streamline genetic engineering in *Synechococcus* sp. PCC 7002, we validated three NISs that are regularly used in *Synechococcus*; henceforth noted as A2842, A0935 and A0159. Begemann et al. showed that the insertion of DNA fragments in *glpK* (*SYNPCC7002_A2842*) has no influence on the physiology of *Synechococcus* [23]. *SYNPCC7002_A2842* has previously been annotated as a pseudogene due to a frameshift in the genetic sequence. However, recent research suggests that the presumed frameshift is due to a sequencing error, indicating that *glpK* is still intact in the genome of *Synechococcus* [32]. The NIS noted as A0935 is based on a study by Davies et al. who showed that insertion of DNA between the two open-reading frames of the hypothetical proteins SYNPCC7002_A0935 and SYNPCC7002_A0936 of *Synechococcus* leads to no adverse change in the growth rate [24]. Sakamoto et al. showed that deletion of *desB* (*SYNPCC7002_A0159*) has no influence on *Synechococcus* growth at temperatures above 22 °C [25] and this site is already regularly used as a NIS [17]. Since *Synechococcus* is routinely grown at 33–38 °C, the gene *SYNPCC7002_A0159* is assumed a NIS when standard conditions are met [25]. Based on these previous studies, we expected that insertion of DNA into the three NISs would not have an adverse effect on growth. In order to replicate the use of the NIS as closely as possible, we decided to use the same gene insertion approach as used in previous studies. Figure 1 provides a complete overview of the genetic context and gene insertion approaches used in our study.

### Assessment of transformation efficiency

Natural transformation in *Synechococcus* relies on the presence of homologous regions between the chromosome and the dsDNA that is transferred. We were therefore interested in how the length of the homologous region of the dsDNA would influence the success of transformation. Transformation protocols for *Synechococcus* use homologous regions that vary from 250 to 1000 nt [33–35]. Since synthesizing homologous regions is a crucial cost factor, we assessed which minimal length of homologous regions still results in a suitable high number of transformants. To study the influence of the homologous regions on transformation success, we designed four genetic modules for each of the three NISs A2842, A0159 and A0935. Each construct contained either 800, 400, 200 or 100 nt homologous region of the targeted NIS and a kanamycin resistance cassette flanked by BioBrick Prefix and Suffix. We have used the BioBrick system since it allows us to exchange the kanamycin resistance cassette in our template vectors with any other genetic cassette, thereby enabling us to alter the vector effortlessly for future synthetic biology applications. Our transformation protocol is based on previous studies on *Synechocystis* sp. PCC 6803, summarized by Eaton-Rye [20]. Since Stevens and Porter showed that transformation during the exponential growth phase leads to the highest transformation success [19], we used *Synechococcus* cultures with an OD_730_ of 0.4 for transformation [26]. The dsDNA constructs were inserted into the genomic DNA of *Synechococcus* by homologous recombination, and the resulting transformants were plated onto selective agar plates. Transformation efficiency was assessed with two different approaches: (i) Optical density at 730 nm after 7 days of growth and (ii) intensity of spotted *Synechococcus* cultures with a camera-based plate imaging system (manuscript in preparation) after 12 days of growth.

### Assessment of transformation efficiency using a spectrophotometer

**Fig. 2:**
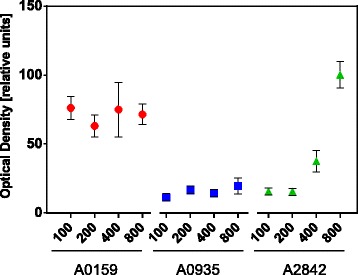
Results of the transformation efficiency assessment by optical density measurements at 730 nm. *Synechococcus* sp. PCC 7002 strains were transformed with a kanamycin resistance cassette containing either 100, 200, 400 or 800 nt homology with the targeted genetic site in the chromosome to induce homologous recombination. Three different approaches were used to target three sites: (1) Insertion in the non-coding region between *SYNPCC7002_A0935* and *SYNPCC7002_A0936* (noted as A0935), (2) gene disruption of the coding region of *desB* (noted as A0159) and (3) gene replacement of *glpK* (noted as A2842). The optical density at 730 nm (OD_730_) of the constructed mutants were measured after transformation and antibiotic selection and normalized to the highest measured OD_730_. Data points are the average of three separate experiments, and the error bars are indicative of the standard error

To assess the transformation efficiency without introducing plating efficiency as a factor, all colonies resulting from transformation and subsequent antibiotic selection were resuspended in AA+ medium and quantified by spectrophotometric OD_730_ measurements (Additional file 5: Figure S4). A sample was taken to continue strain generation in order to confirm the successful transformation by colony PCR after segregation (Additional file 6: Figure S5). The OD_730_ was normalized to the highest OD of the colony dilutions (Fig. 2). Our data indicates that we achieved successful transformations for all the three NIS even with homologous regions as short as 100 nt. However, the total number of successful chromosomal integrations differs. In addition, the data show that the lowest transformation success is observed for A0935. There is a correlation between the transformation efficiency and the length of the homologous regions for A2842. For A0159, transformation efficiency seems to be independent of the homologous region length. To confirm our findings and strengthen our hypothesis, we performed an additional, independent transformation experiment and utilized a camera-based plate imaging system to assess transformation efficiency.

### Assessment of transformation efficiency using a camera-based plate imaging system

**Fig. 3:**
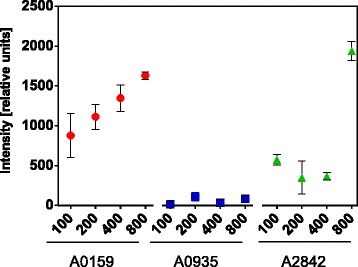
Results of the transformation efficiency assessment by camera-based plate imaging. *Synechococcus* sp. PCC 7002 strains were transformed with a kanamycin resistance cassette containing either 100, 200, 400 or 800 nt homology with the targeted genetic site in the chromosome to induce homologous recombination. Three different approaches were used to target three sites: (1) Insertion in the non-coding region between *SYNPCC7002_A0935* and *SYNPCC7002_A0936* (noted as A0935), (2) gene disruption of the coding region of *desB* (noted as A0159) and (3) gene replacement of *glpK* (noted as A2842). The intensities of spotted transformant colonies on plates of the constructed mutants were measured after transformation and antibiotic selection and analyzed with a camera-based method. Data points are the average of three separate experiments, and the error bars are indicative of the standard error

To confirm different transformation efficiencies obtained by the spectrophotometric measurements, we repeated the transformation and used a plate imaging system to quantify transformation efficiencies. For this, we spotted batches of cells that have undergone the transformation procedure onto selective plates and assessed the increase of density of these spots during 12 days (Additional file 7: Figure S6). We used the data points of the last measurement to compare the transformation success for each of the constructs (Fig. 3). These data show that A0935 has the lowest transformation efficiency. Homologous regions of 800 nt result in the highest transformation success in A2842 but a correlation between transformation efficiency and length of homologous regions similar to the spectrophotometric measurements is not clearly visible. It seems that transformation success of A1059 has a slight dependence on the lengths of the homologous regions; however, this effect is not pronounced.

### Parameters influencing transformation efficiency

The results of both (spectrophotometric and camera-based) experiments show that for the three NISs, a high number of transformants could be achieved and modified strains could be successfully segregated. The strains generated were used for subsequent growth experiments. Both sets of experiments reveal a distinct difference in transformation efficiency depending on the location of the integration sites within the genome. The lengths of the homologous regions seem to influence the transformation efficiency in A2842 and to some extent in A0159, but has no detectable influence in A0935. Ruffing et al. also showed a correlation between the length of the homologous regions and transformation success for the NIS A0159 [17]. However, in Ruffing’s study, successful transformation could not be achieved with homologous regions shorter than 250 nt. In contrast, here we report successful transformation with homologous regions as short as 100 nt with a higher transformation efficiency (based on calculations with 6 h of incubation) for constructs targeting A0159 (Additional file 8: Figure S7). Previously, Davies et al. also used the location between the genes *SYNPCC7002_A0935* and *SYNPCC7002_A0936* for their study using 750 nt homologous regions. We can therefore conclude that A0935 can be utilized as a NIS, albeit at a lower transformation efficiency compared to A0159 or A2842. Thus, it is advisable to use A0159 or A2842 and to avoid using A0935 as a site for integration of non-native DNA in order to obtain a high number of transformants. The observed transformation efficiency is influenced by many factors including the rate of DNA uptake; degradation of DNA outside and inside of the cell; and the rate of successful integration of DNA into the host genome. It has previously been shown that the success of DNA integration into the genome of *Synechococcus* is decreasing with increasing lengths of integrated non-native DNA [36]. However, in our study, the length of non-native DNA is constant due to comparable homologous regions and the use of the same antibiotic cassette (Additional file 1: Figure S1, Additional file 2: Figure S2 and Additional file 3: Figure S3). The length of DNA fragments used for transformation can therefore be excluded as a factor for our results. Analysis of transcriptional data in *Synechococcus* [26] reveals that both *SYNPCC7002_A0935* and *SYNPCC7002_A0936* are only transcribed at low levels under standard conditions, just like neighboring genes, thereby excluding a negative effect of homologous recombination on essential (neighboring) genes. Instead, intracellular processes, such as nucleolytic processing and dependency of the recombination frequency based on genomic position [36–39], are probably the main causes for the differences in efficiency of homologous recombination in our study. Additionally, a low transformation efficiency can be caused by factors impairing homologous recombination, such as secondary DNA structures making parts of the chromosome more accessible than other parts [40]. It is further possible that the intergenic region between *SYNPCC7002_A0935* and *SYNPCC7002_A0936* contains genetic structures and functions that are not yet annotated. Previous research on transformation in other cyanobacteria already revealed a correlation between genomic position and recombination frequency [39, 40].

### Growth analysis

**Fig. 4:**
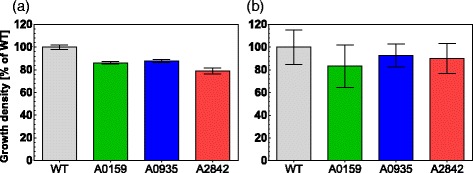
Growth densities of *Synechococcus* sp. PCC 7002 wildtype and A0159, A0935 and A2842 strains. Growth density was calculated based on data obtained either by spectrophotometric measurements or by measuring spot intensities and related to the *Synechococcus* sp. PCC 7002 wildtype growth density. Statistical tests were performed with the unpaired t-test provided by Prism version 6 (GraphPad). Shown are the average of technical triplicates and *error bars* indicate the standard error. **a** Maximal growth density of A0159, A0935 and A2842 strains as a percentage of wildtype density in liquid medium. Mutated strains and wildtype *Synechococcus* sp. PCC 7002 were grown in liquid medium and OD_730_ measurements were performed every 24 h. Two-tailed *P*-values were 0.0018 (A2842), 0.0060 (A0935) and 0.0034 (A0159). **b** Maximal growth density A0159, A0935 and A2842 strains as a percentage of wildtype density on solid medium. Cultures of *Synechococcus* strains A0159, A0935 and A2842 were spot-plated and the relative change in intensity of the spots was assessed by a camera-based method. Wildtype *Synechococcus* sp. PCC 7002 was used as a control

Comparative growth experiments under standard growth condition reveal that insertion of a kanamycin cassette at A2842, A0935 and A0159 results in a slightly but significantly lower growth rate compared to wildtype *Synechococcus* in liquid medium (Fig. 4a). This is probably due to a fitness cost caused by the expression of the kanamycin resistance gene [41]. However, this fitness cost is not clearly observable during growth on solid medium (Fig. 4b), which may be due to the quality of the data. Growing A0159, A0935 and A2842 on a kanamycin concentration of 50 μg/mL or 100 μg/mL did not reveal differences in growth. This result indicates that the kanamycin resistance cassette is not expressed at different levels within the different NISs. It should be noted however that our growth experiments were conducted under standard conditions as defined in the Method section. Growing the strains A0159, A0935 and A2842 under non-standard conditions may result in different growth behavior.

### Further optimization of the transformation

Since transformation of A0159 and A2842 with 100 nt homologous regions leads to a high number of transformants, it may be possible to use shorter flanking region for successful transformations, such as seen for *Escherichia coli* (36–50 nt) or *Saccharomyces cerevisiae* (35–51 nt) [42, 43]. Additionally, it would be valuable to investigate if the length of the insert of exogenous DNA has any effect on transformation efficiency. Previous studies indicate that an increase of 2–3 kb in exogenous DNA insert length decreases the overall transformation frequency by half [36]. For the identification of additional NISs in *Synechococcus*, a systematical approach as implemented by Pinto et al. for *Synechocystis* sp. PCC6803 is advisable [1].

The transformation protocol we developed during this study [27] has some improvement over previously reported protocols. The overall time required to obtain segregated mutants with our protocol is estimated to be at least 12–19 days (Additional file 4: Table S2) shorter compared to the previous transformation protocol reported for *Synechococcus* [21], assuming that segregation takes the same amount of time for both protocols. This almost 50% reduction is accomplished without the use of elevated CO_2_ concentrations that accelerate growth in the reference protocol. In contrast to Frigaard et al., we do not bubble the cultures with air or shake cultures during incubation with exogenous DNA. This allows DNA to interact with the cell surface, thus aiding pilin-facilitated DNA uptake. The most likely reason for the improvements in time and number of transformants is that we do not use top agar to expose the cells to antibiotic selection. The top agar may slow down growth of *Synechococcus*. Instead, we use sterile membranes to transfer cells to antibiotic-containing plates, a method adapted from *Synechocystis* sp. PCC6803 transformations [20]. Additionally, our transformation method uses linear DNA fragments for transformation instead of plasmids. Since these fragments can be either ordered from commercial parties or constructed with standard PCR, our protocol skips the time-consuming and costly step of constructing and ordering transformation plasmids.

Stevens and Porter showed that the incubation optimum on non-selective plates is reached after eight doubling times [19]. Since *Synechococcus* had a doubling time of about 7 h under our tested conditions, it is possible that the amount of successful transformants can be increased by longer incubation on non-selective plates. In addition, we know from previous experiments that the incubation times can be shortened, for example by immediately plating on selective plates or by lowering the initial OD_730_. However, this has a negative effect on the number of transformants (unpublished data).

## Conclusions

In this study, we present an improved transformation protocol for *Synechococcus* sp. PCC 7002 and BioBrick-compatible modules that allow the integration of genetic constructs into the NISs A2842, A0159 and A0935 with homologous regions as short as 100 nt. The BioBrick-compatible modules can easily be modified to integrate different DNA fragments. Since homologous regions as short as 100 nt can be used to insert DNA into the NISs, other, non-cloning-based approaches are also feasible. The combination of long primers containing homologous regions with standard PCR of the insertion fragment provides an effective way to create linear dsDNA for transformation at low costs. Additionally, commercial parties offer the synthesis of dsDNA fragments at low costs and with short delivery times. This study contributes to developing *Synechococcus* as the prominent chassis for green synthetic biology and enabling high throughput genetic engineering techniques.

## Additional files

Additional file 1: Figure S1. Genetic constructs for transformation of A2842. (a) Plasmid map of pUC57Simple_A2842Kan. (b)–(e) Schematic overview of the DNA fragments with either 800, 400, 200 or 100 nt, used for transformations. The scale is indicated under (e). The kanamycin-BioBrick cassette is indicated in blue and the homologous regions in gray. The resulting genetic organization is displayed in Fig. 1b. (PDF 80 kb)

Additional file 2: Figure S2. Genetic constructs for transformation of A0935. (a) Plasmid map of pUC57Simple_A0935Kan. (b)–(e) Schematic drawing of the DNA fragments with either 800, 400, 200 or 100 nt resp. used for transformation. The homologous regions are indicated in gray while the kanamycin-BioBrick cassette is indicated in blue. The scale is indicated under (e). The transformation product resulting from these fragments is displayed in Fig. 1d. (PDF 77 kb)

Additional file 3: Figure S3. Genetic constructs for transformation of A0159. (a) Plasmid map of pUC57Simple_A0159Kan. A detailed map can be found at the Benchling Plasmid Repository [29]. (b)–(e) Schematic representation of the DNA fragments used for transformation with resp. 800, 400, 200 or 100 nt homologous regions. The homologous regions are indicated in gray and the kanamycin-BioBrick cassette in blue. The scale is indicated under (e). The resulting transformation product is depicted in Fig. 1f. (PDF 72 kb)

Additional file 4: Tables S1 and S2. Overview of primers used in this study. The primers were used for generating linear dsDNA fragments with different lengths of homologous regions. The name indicates the neutral integration site, the length of the homologous region and the direction of the primer. **Table S2**. Comparison of the time required for the different transformation steps in the protocol provided by Frigaard et al. [21] versus the newly developed transformation protocol based on our research. The numbers indicate hours. (PDF 285 kb)

Additional file 5: Figure S4. Cell number plotted against optical density at 730 nm based on cell counting. In order to verify the correlation between OD and cell number, we performed cell counting experiments against the measured OD730 for each generated strain. For the wildtype Synechococcus strain and the generated Synechococcus strains A2842, A0935 and A0159, cultures were grown under standard conditions until OD730 of 1. Cultures were diluted in triplicate to an OD730 of approximately 0.1 and 0.2. OD730 was verified with spectrophotometry. Cell numbers were determined with the BD Accuri FlowCytometer C6 (BD-Bioscience, Germany), with a C-Sampler according the manual. The relation between the number of cells and the OD730 was plotted with Prism version 6 (GraphPad). No significant difference is apparent between the wildtype Synechococcus strain and the three NSI mutant strains (Ordinary one-way ANOVA, *P*-value: 0.6129). (PDF 18 kb)

Additional file 6: Figure S5. Colony PCR results. (a) Colony PCR results for two potential Synechococcus A2843 strains (lane 1–2) with a wildtype Synechococcus strain (lane 3) as control. The primers A2842-800-Fwd and A2842-800-Rv were used. The result in lane 1 shows that this A2843 strain is not yet fully segregated, while the results shown in lane 2 indicate that full segregation took place. (b). Results for colony PCR with the primers A0935-800-Fwd and A0935-800-Rv. Three potential Synechococcus A0935 strains were used as template (lane 1–3), as well as a Synechococcus wildtype strain as a control (lane 4). The results indicate that the first A0935 strain is fully segregated (lane 1), while a additional band indicates the presence of the wildtype gene (non-segregated strain) lane 3. (c) The results of the colony PCR performed with the primers A0159-800-Fwd and A0159-800-Rv and three potential Synechococcus A0159 strains show that all three tested strains are fully segregated (lane 1–3). Wildtype Synechococcus was used as a control (lane 4). (PDF 56 kb)

Additional file 7: Figure S6. Assessment of transformation efficiency using a camera-based plate imaging system. Pictures were taken every 8 h. The change in intensity during 280 h is depicted for three different neutral integration sites A0159 (a), A0935 (b) and A2842 (c) in Synechococcus. The length of the homologous regions used during transformation is indicated in the legend behind the strain name. (PDF 83 kb)

Additional file 8: Figure S7. Transformation efficiency based on assessment by optical density measurements at 730 nm. Synechococcus sp. PCC 7002 strains were transformed with genetic modules containing a kanamycin resistance cassette and either 100, 200, 400 or 800 nt homologous regions. The three neutral integration sites A0159, A0935 and A2842 were targeted. The optical density at 730 nm (OD730) of the constructed mutants were measured after successful transformation. Transformation efficiency was calculated by calculating cell numbers based on the OD730 and dividing the number of successful transformants by the fmol DNA used. (PDF 41 kb)
