# Supplementary figures and images for: Streamlining recombination-mediated genetic engineering by validating three neutral integration sites in Synechococcus sp. PCC 7002
Source: J Biol Eng. 2017 Jun 5;11:19. doi: 10.1186/s13036-017-0061-8 (PMC5458483; doi:10.1186/s13036-017-0061-8)

(a)

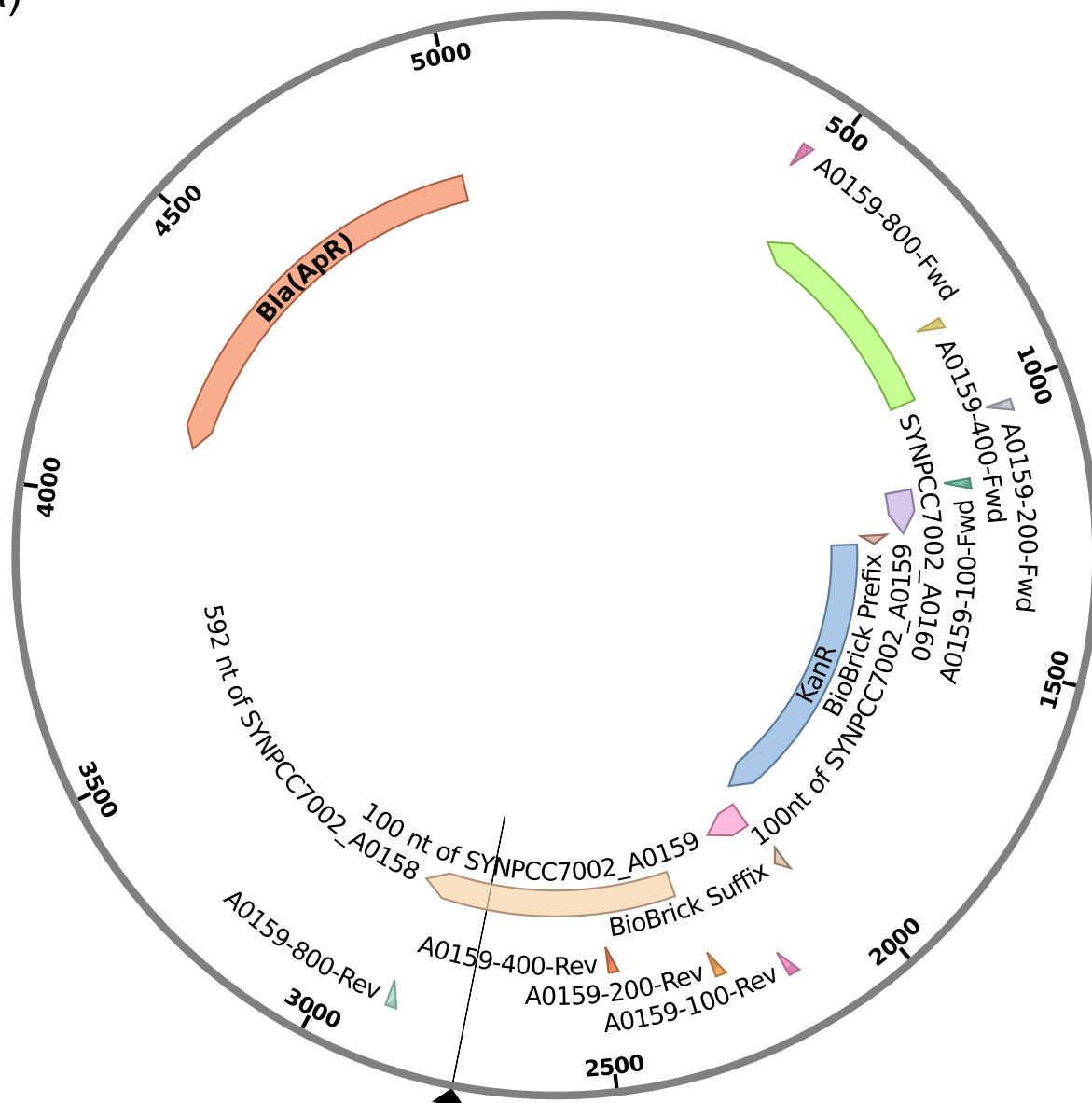

(b)

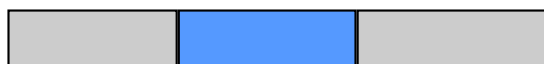

(c)

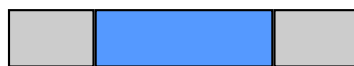

(d)

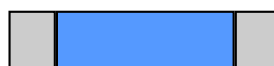

(e)

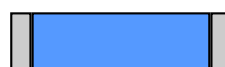

500 nt

Supplement: Supplementary file 1 — Genetic constructs for transformation of A2842. (a) Plasmid map of pUC57Simple_A2842Kan. (b)–(e) Schematic overview of the DNA fragments with either 800, 400, 200 or 100 nt, used for transformations. The scale is indicated under (e). The kanamycin-BioBrick cassette is indicated in blue and the homologous regions in gray. The resulting genetic organization is displayed in Fig. 1b. (PDF 80 kb) [file 13036_2017_61_MOESM1_ESM.pdf]

(a)

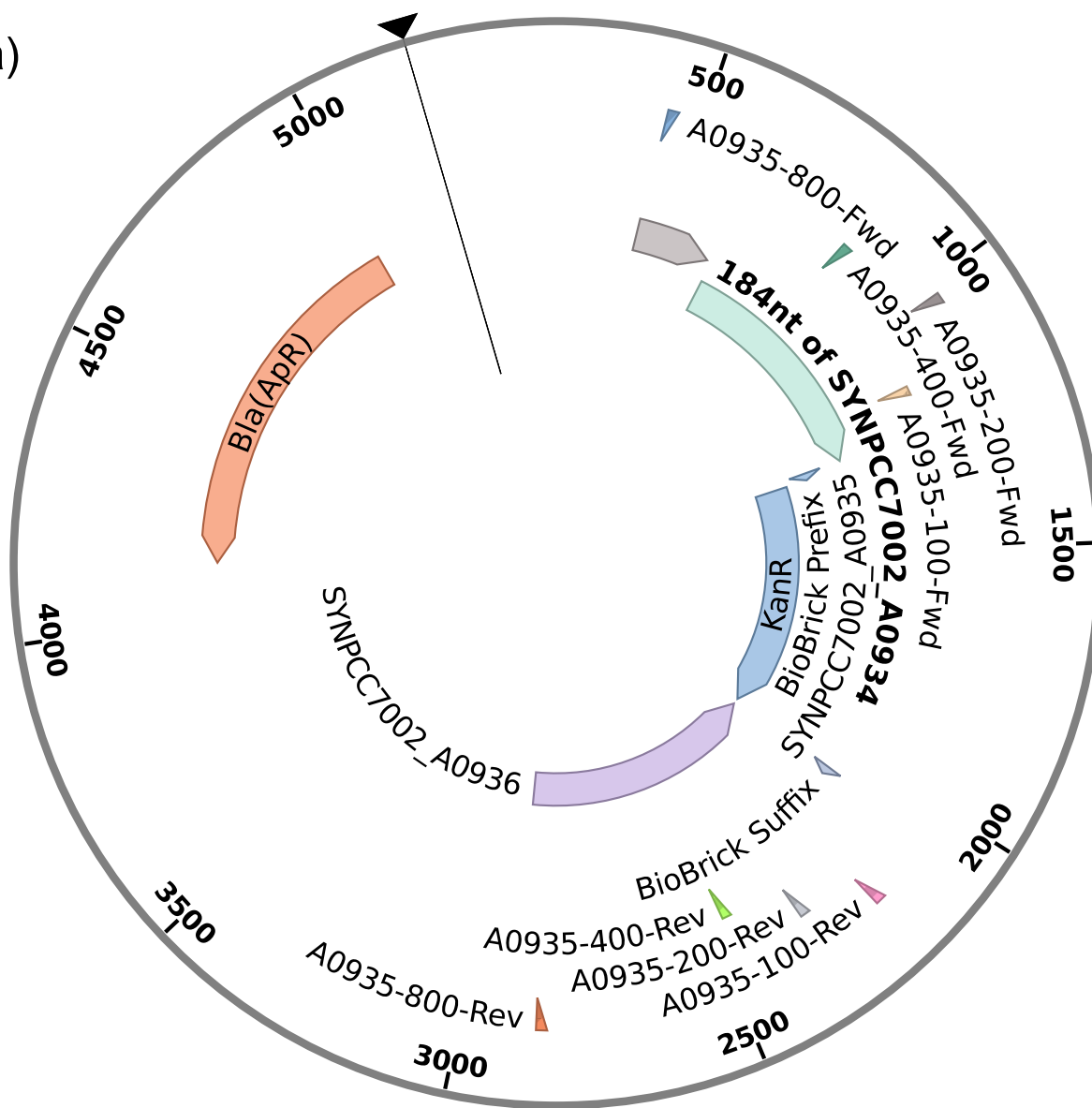

(b)

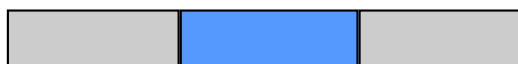

(c)

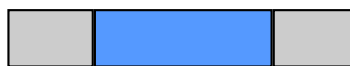

(d)

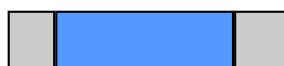

(e)

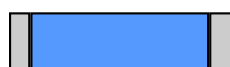

500 nt

Supplement: Supplementary file 2 — Genetic constructs for transformation of A0935. (a) Plasmid map of pUC57Simple_A0935Kan. (b)–(e) Schematic drawing of the DNA fragments with either 800, 400, 200 or 100 nt resp. used for transformation. The homologous regions are indicated in gray while the kanamycin-BioBrick cassette is indicated in blue. The scale is indicated under (e). The transformation product resulting from these fragments is displayed in Fig. 1d. (PDF 77 kb) [file 13036_2017_61_MOESM2_ESM.pdf]

(a)

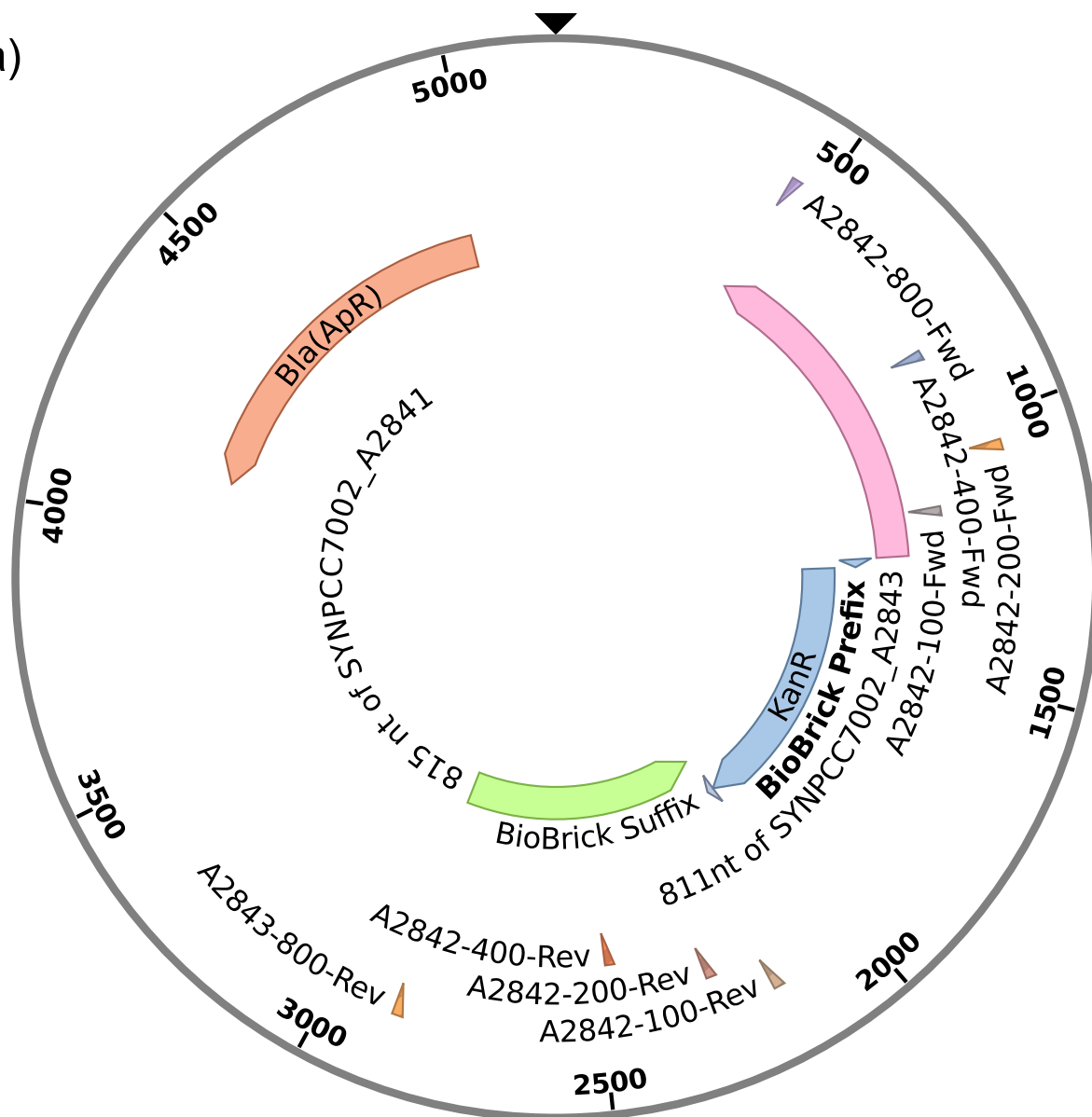

(b)

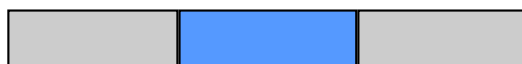

(c)

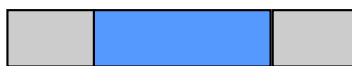

(d)

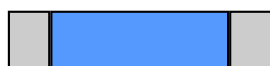

(e)

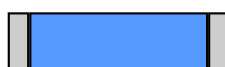

500 nt

Supplement: Supplementary file 3 — Genetic constructs for transformation of A0159. (a) Plasmid map of pUC57Simple_A0159Kan. A detailed map can be found at the Benchling Plasmid Repository [29]. (b)–(e) Schematic representation of the DNA fragments used for transformation with resp. 800, 400, 200 or 100 nt homologous regions. The homologous regions are indicated in gray and the kanamycin-BioBrick cassette in blue. The scale is indicated under (e). The resulting transformation product is depicted in Fig. 1f. (PDF 72 kb) [file 13036_2017_61_MOESM3_ESM.pdf]

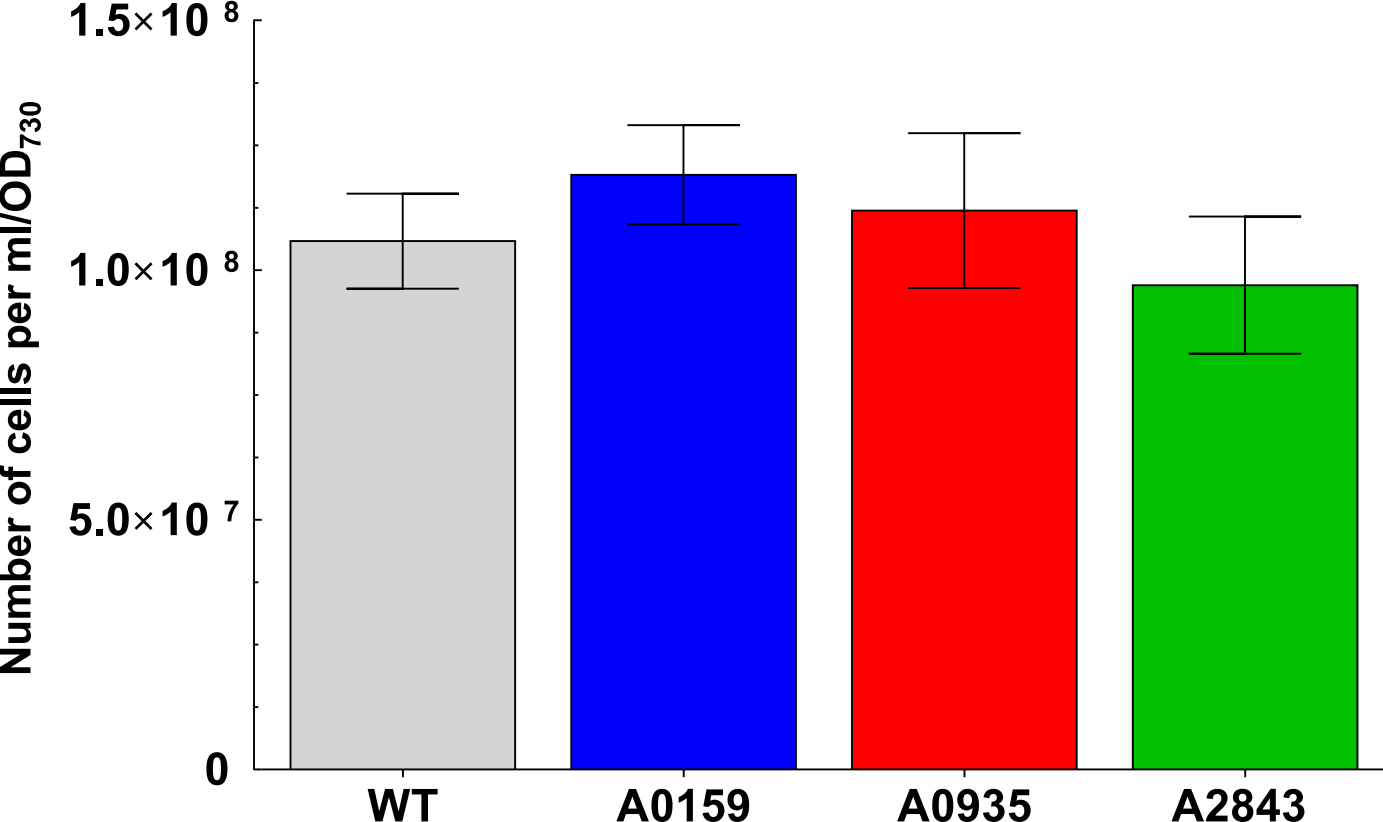

Supplement: Supplementary file 5 — Cell number plotted against optical density at 730 nm based on cell counting. In order to verify the correlation between OD and cell number, we performed cell counting experiments against the measured OD730 for each generated strain. For the wildtype Synechococcus strain and the generated Synechococcus strains A2842, A0935 and A0159, cultures were grown under standard conditions until OD730 of 1. Cultures were diluted in triplicate to an OD730 of approximately 0.1 and 0.2. OD730 was verified with spectrophotometry. Cell numbers were determined with the BD Accuri FlowCytometer C6 (BD-Bioscience, Germany), with a C-Sampler according the manual. The relation between the number of cells and the OD730 was plotted with Prism version 6 (GraphPad). No significant difference is apparent between the wildtype Synechococcus strain and the three NSI mutant strains (Ordinary one-way ANOVA, P-value: 0.6129). (PDF 18 kb) [file 13036_2017_61_MOESM5_ESM.pdf]

(a)

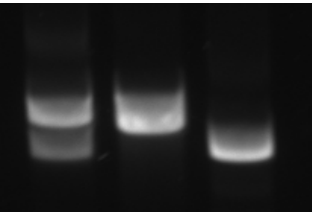

(b)

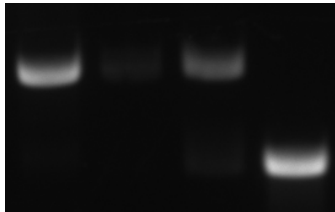

(c)

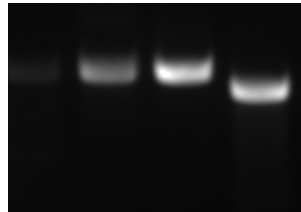

Supplement: Supplementary file 6 — Colony PCR results. (a) Colony PCR results for two potential Synechococcus A2843 strains (lane 1–2) with a wildtype Synechococcus strain (lane 3) as control. The primers A2842-800-Fwd and A2842-800-Rv were used. The result in lane 1 shows that this A2843 strain is not yet fully segregated, while the results shown in lane 2 indicate that full segregation took place. (b). Results for colony PCR with the primers A0935-800-Fwd and A0935-800-Rv. Three potential Synechococcus A0935 strains were used as template (lane 1–3), as well as a Synechococcus wildtype strain as a control (lane 4). The results indicate that the first A0935 strain is fully segregated (lane 1), while a additional band indicates the presence of the wildtype gene (non-segregated strain) lane 3. (c) The results of the colony PCR performed with the primers A0159-800-Fwd and A0159-800-Rv and three potential Synechococcus A0159 strains show that all three tested strains are fully segregated (lane 1–3). Wildtype Synechococcus was used as a control (lane 4). (PDF 56 kb) [file 13036_2017_61_MOESM6_ESM.pdf]

(a)

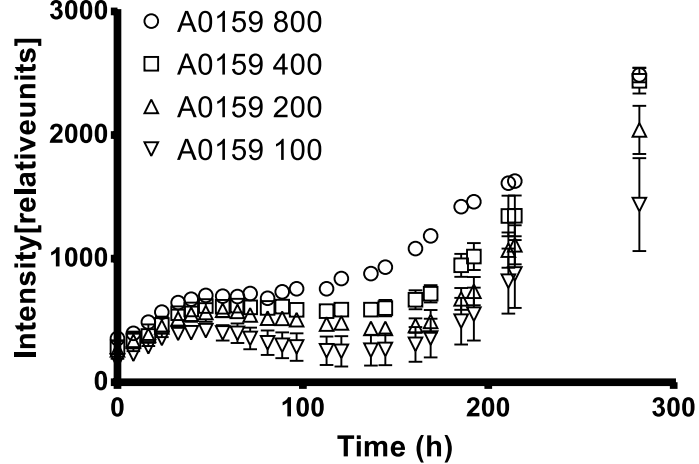

(b)

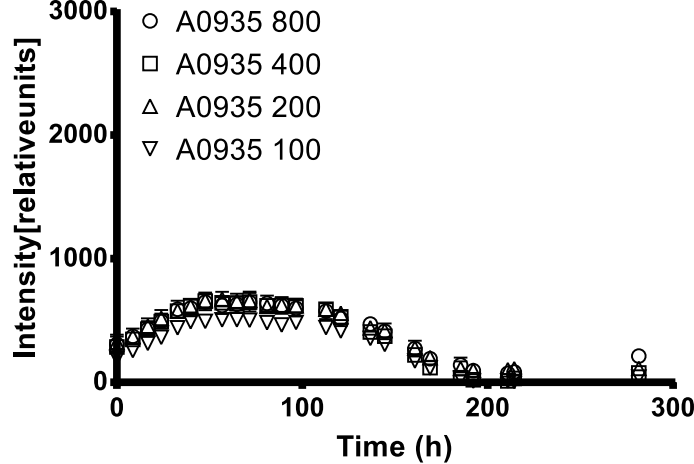

(c)

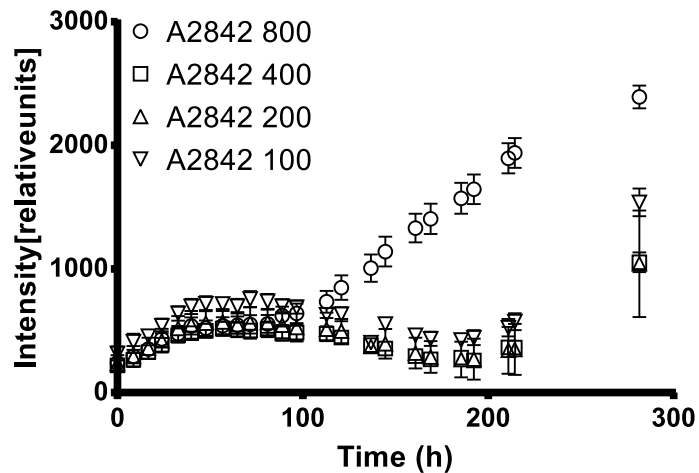

Supplement: Supplementary file 7 — Assessment of transformation efficiency using a camera-based plate imaging system. Pictures were taken every 8 h. The change in intensity during 280 h is depicted for three different neutral integration sites A0159 (a), A0935 (b) and A2842 (c) in Synechococcus. The length of the homologous regions used during transformation is indicated in the legend behind the strain name. (PDF 83 kb) [file 13036_2017_61_MOESM7_ESM.pdf]

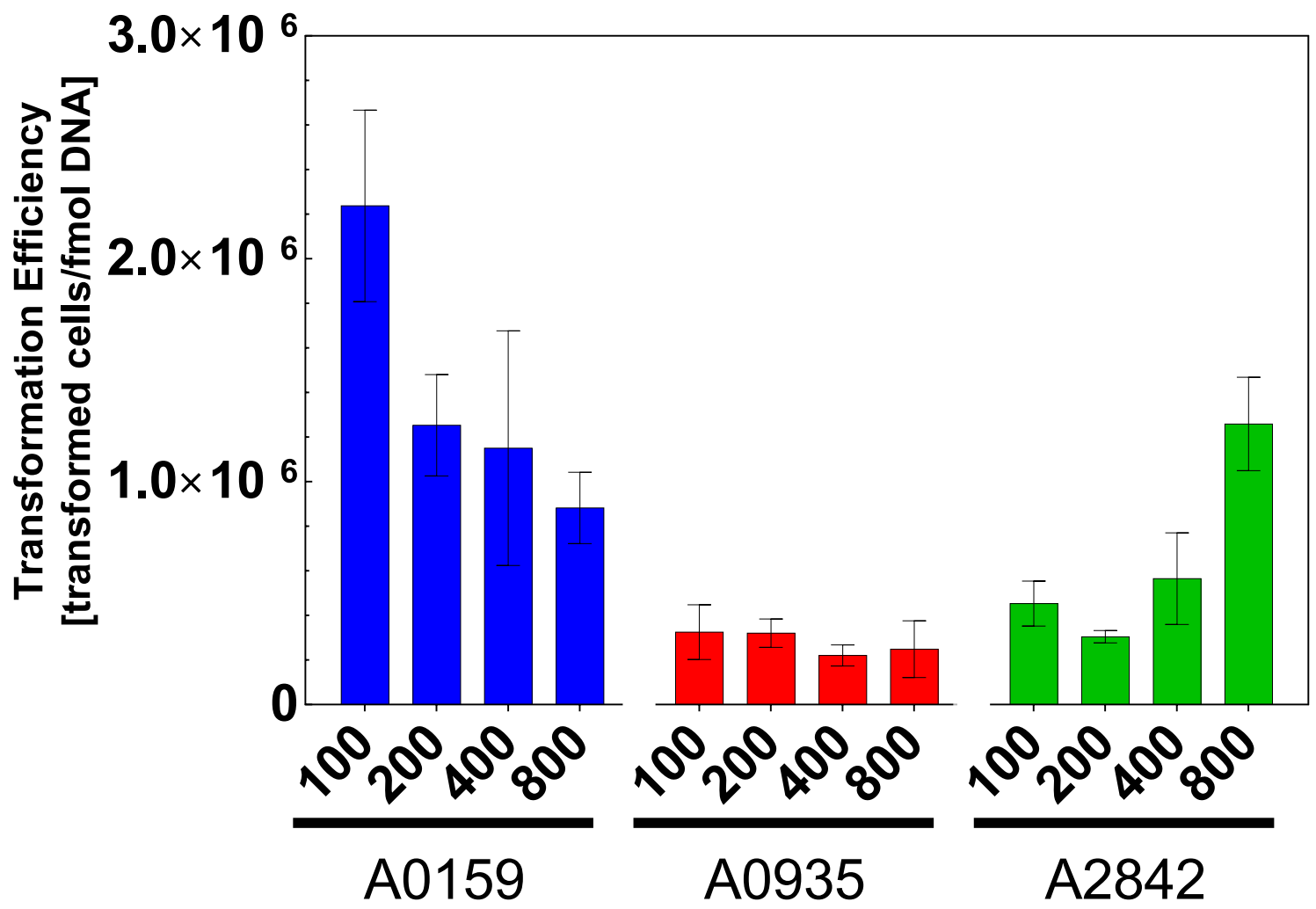

Supplement: Supplementary file 8 — Transformation efficiency based on assessment by optical density measurements at 730 nm. Synechococcus sp. PCC 7002 strains were transformed with genetic modules containing a kanamycin resistance cassette and either 100, 200, 400 or 800 nt homologous regions. The three neutral integration sites A0159, A0935 and A2842 were targeted. The optical density at 730 nm (OD730) of the constructed mutants were measured after successful transformation. Transformation efficiency was calculated by calculating cell numbers based on the OD730 and dividing the number of successful transformants by the fmol DNA used. (PDF 41 kb) [file 13036_2017_61_MOESM8_ESM.pdf]
