# Supplementary material for: Streamlining recombination-mediated genetic engineering by validating three neutral integration sites in Synechococcus sp. PCC 7002
Source: J Biol Eng. 2017 Jun 5;11:19. doi: 10.1186/s13036-017-0061-8 (PMC5458483; doi:10.1186/s13036-017-0061-8)
Supplement: Supplementary file 4 — Overview of primers used in this study. The primers were used for generating linear dsDNA fragments with different lengths of homologous regions. The name indicates the neutral integration site, the length of the homologous region and the direction of the primer. Table S2. Comparison of the time required for the different transformation steps in the protocol provided by Frigaard et al. [21] versus the newly developed transformation protocol based on our research. The numbers indicate hours. (PDF 285 kb) [file 13036_2017_61_MOESM4_ESM.pdf]

Tables

Table S1: Overview of primers used in this study. The primers were used for generating linear dsDNA fragments with different lengths of homologous regions. The name indicates the neutral integration site, the length of the homologous region and the direction of the primer.

Name	Sequence (5' - 3')
A0935-800-Fwd	AGTTTCCGGATCGATGAACC
A0935-400-Fwd	CGCCCTTCCTTTGTGCTCAG
A0935-200-Fwd	ACCAATCTTCAGCGGAAACAG
A0935-100-Fwd	ACGGGCTTCTAGCACAAATG
A0935-800-Rev	AATTGAGACTTCAATTTATC
A0935-400-Rev	CTGTTTACAGCAGCCCTTAG
A0935-200-Rev	ACCACGCCAATGTCTACACC
A0935-100-Rev	CGGTACAGATGTCTTGATCG
A2842-800-Fwd	CGATAATCCAGCAACCCAAC
A2842-400-Fwd	TTATGGAGGATGGCCACGAG
A2842-200-Fwd	CCAGCAGCGGCTTGTGTTAG
A2842-100-Fwd	CGATCGCCACACCGACTTTG
A2842-800-Rev	TAGTAATAATTCAGCAGCAC
A2842-400-Rev	AATCTGGCACTGTGGCAAGG
A2842-200-Rev	CGTTGGTGAGGACAAAGAAG
A2842-100-Rev	TCTAGATCTTGAATAATGAG
A0159-800-Fwd	CCGATTAGACCCTAAATTTC
A0159-400-Fwd	TACCGCCAGGATCGGAAAGC
A0159-200-Fwd	ATCGCCAGGGCCAAACAATG
A0159-100-Fwd	ATGCAAAGTACAGTCCGCTC
A0159-800-Rev	TTTCTGCAAGGCGTTGTTGG
A0159-400-Rev	CACCAGGGCTAACACTTCAG
A0159-200-Rev	TCTCGATCATAGGCGATCTC
A0159-100-Rev	CGGCTGGTAATACAAGTGAG

Table S2: Comparison of the time required for the different transformation steps in the protocol provided by Frigaard *et al.* [21] versus the newly developed transformation protocol based on our research. The numbers indicate hours.

	Incubation with DNA	Incubation (non-selective plates)	Incubation (selective plates)	Segregation	Total
Frigaard *et al.*	5	72	336-504	192	605-773
This work	6	24	96	192	318
